# Roles of elements V and N in tetragonally distorted Fe-Co-V-N films

**DOI:** 10.1038/s41598-025-13671-3

**Published:** 2025-08-05

**Authors:** Takashi Hasegawa, Chihiro Murakami, Kosuke Imamura, Yuta Nakamura, Mitsuru Ohtake, Haruki Yamane

**Affiliations:** 1 Akita University, Akita, Japan; 2 Yokohama National University , Yokohama, Japan; 3 Akita Industrial Technology Center , Akita, Japan

**Keywords:** Lattice distortion, Tetragonal distortion, Uniaxial magnetic anisotropy, XRD, Materials for devices, Electrical and electronic engineering, Magnetic properties and materials

## Abstract

Tetragonally distorted Fe-Co-V-N with a body-centred tetragonal (bct) structure has a high saturation magnetisation and a high magnetocrystalline anisotropy of ~ 1 MJ/m^3^ and is expected to be a new hard magnetic material. The effectiveness of the combined addition of the elements V and N for bct formation has been reported, but the mechanism remains unclear. In this study, the amounts of V and N in FeCo were varied systematically, and the optimal amounts were found to be approximately 20 at% V and 6 at% N. This optimal composition was also effective for the bct transformation, even in a 100-nm-thick film. The role of V in the bct transformation is assumed to be to lower the energy needed to change the lattice constant of FeCo and attract N. The role of N is also considered to extend the *c*-axis of the Fe-Co-V lattice, causing a bct transformation.

## Introduction

According to the Slater–Pauling curve, FeCo has the highest saturation magnetisation (*M*_s_) among all the transition-metal alloys^1^. FeCo has a body-centred cubic (bcc) structure with an axial ratio (*c*/*a*) of 1.00 at room temperature, and its Curie temperature is approximately 980 K, which is approximately three times that of Nd-Fe-B magnets. However, FeCo with a bcc structure has an extremely small uniaxial magnetic anisotropy constant (*K*_u_) and is therefore known as a typical soft magnetic material. Theoretical calculations have predicted that FeCo transforms into a body-centred tetragonal (bct) structure (1.00 < *c*/*a* < 1.41) that achieves a high *K*_u_ of 10 MJ/m^3^ at *c*/*a* = 1.25^2,3^. The following two methods have been investigated for the experimental synthesis of bct FeCo.

(i) Utilising the lattice mismatch between the underlayer and the FeCo layer (which is epitaxially grown).
(ii) Stabilising the bct phase by adding a third element to FeCo.

In method (i), the compressive stress due to lattice mismatch in extremely thin FeCo (thickness *t* < ~ 5 nm) epitaxially grown on various underlayers (Pt^4^, CuAu^5^, FePt^6^, and Rh^7,8^ is used to experimentally reduce the lattice constant *a* of FeCo to achieve *c*/*a* = 1.25, resulting in a high *K*_u_ of 1‒2 MJ/m^3^. It has also been reported that a high coercivity of approximately 0.6 T was obtained by microfabricating bct FeCo films to a grain size of ~ 50 nm using electron-beam lithography^8–10^. However, when using method (i), lattice distortion due to lattice mismatch was limited to a film thickness of < 5 nm^10^.

Therefore, to stably synthesise bct FeCo even with film thicknesses (*t*) of ≥ 5 nm, method (ii) was used to explore combinations of additive elements *αβ* in FeCo-*αβ* (*α*: substitutional elements, *β*: interstitial elements). It was reported that a bct structure was stable or metastable in the region of *t* ≥ 5 nm in the combination of *α* = Al^8,9^, Ti^11^, V^12–16^ and *β* = C^12,16^, N^11,13–15^. In particular, the combination of *α* = V and *β* = N is effective for bct formation even in non-epitaxial FeCo films^17^ and rolled FeCo foils^18^. However, the mechanism underlying the effectiveness of the combination of *α* = V and *β* = N for bct formation remains unclear.

The objective of this study was to determine the optimal amounts of V and N added to FeCo to form the bct phase and to clarify the underlying mechanism. First, the effect of the V addition on FeCo was investigated. Rh, whose lattice constant differs significantly from that of FeCo, was selected as the underlayer, and Fe-Co-V with various amounts of V was deposited at various thicknesses using method (i). The purpose of this experiment was to clarify the ranges of the amount of V and thickness for stabilising the bct phase caused by stress due to the lattice mismatch between the Rh underlayer and the Fe-Co-V film. Next, the effect of the combined addition of V and N to FeCo was investigated using method (ii). The amount of added N was systematically changed, resulting in the bcc‒bct‒face-centred cubic (fcc) transformations^13–15^, and the bond energies of each element were analysed. The purpose of this experiment was to identify the elements that preferentially bonded to N atoms. Finally, we examined the roles of V and N in the bct transformation of FeCo.

## Results

### Crystal structure and magnetic properties of Rh/(Fe_0.5_Co_0.5_)_100−*y*_V_*y*_ films

**Fig. 1:**
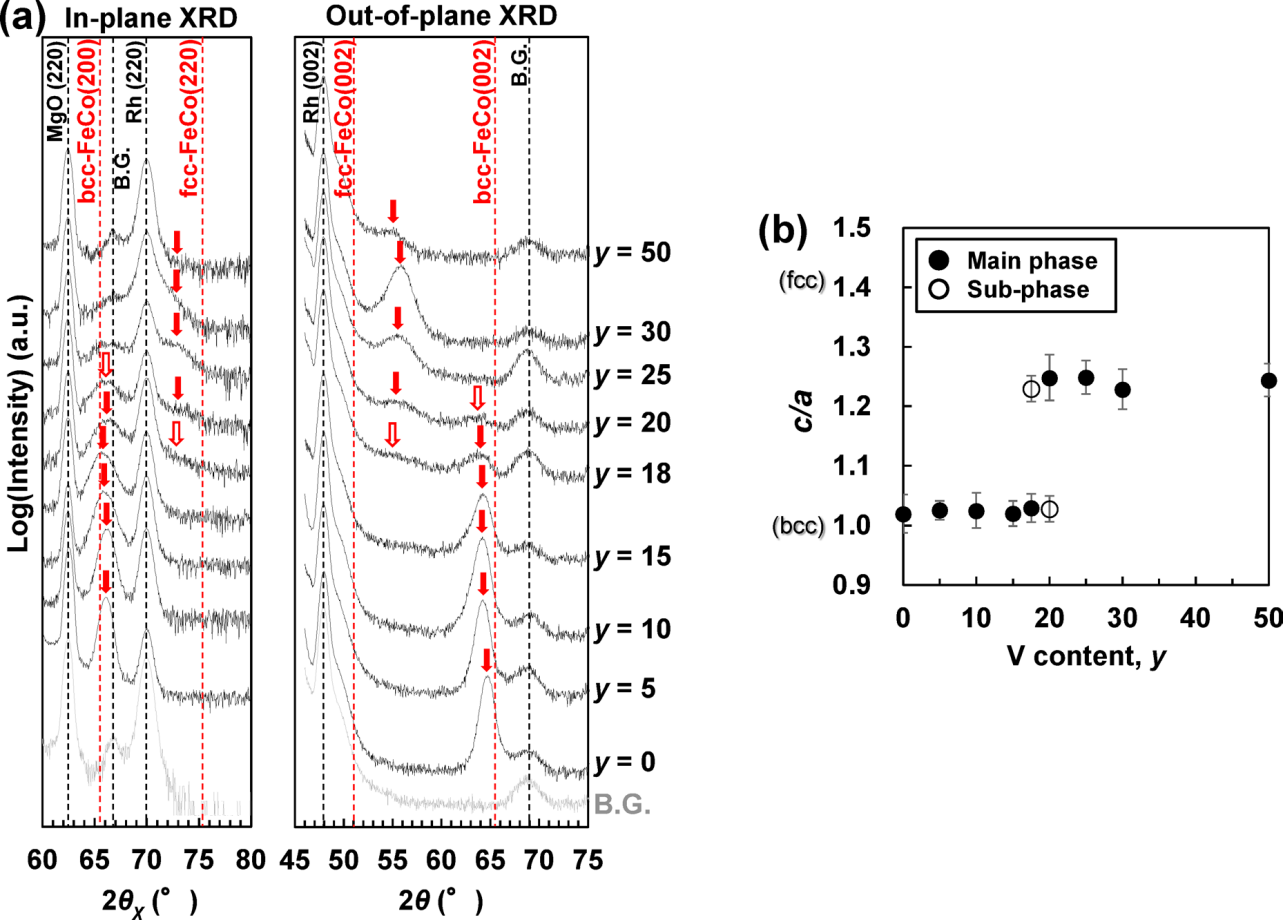
(**a**) In-plane and out-of-plane XRD patterns; (**b**) *y* dependence of *c*/*a* of the MgO sub./Rh (20 nm)/(Fe_0.5_Co_0.5_)_100−*y*_V_*y*_ (20 nm)/SiO_2_ (5 nm) films.

Figure 1a shows the in-plane and out-of-plane X-ray diffraction (XRD) patterns of N-free (Fe_0.5_Co_0.5_)_100−*y*_V_*y*_ (*t* = 20 nm) films deposited on the Rh underlayer. The red solid arrows and hollow arrows indicate the main- and sub-phase peaks derived from the FeCo-based alloy, respectively. Only the peaks from the background (B.G.) of the MgO substrate (sub.)/Rh/SiO_2_ and peaks derived from the FeCo-based alloy were observed, indicating that there were no products other than Fe-Co-V. Additionally, the (001) plane of the Fe-Co-V was oriented perpendicular to the film surface, and the Fe-Co-V was a single-crystal film with the following orientation relationship: MgO (001) [100]//Rh (001) [100]//Fe-Co-V (001) [110] (see inset in Fig. 3b).

In the in-plane XRD patterns, as the V content (*y*) increases, the Fe-Co-V peak—indicated by the red solid arrow—starts from the bcc-FeCo(200) peak position at approximately 65°, passes the Rh(220) peak position at approximately 70°, and shifts to the vicinity of the fcc-FeCo(220) peak position at approximately 75°. This peak shift indicates that the lattice constant *a* of the Fe-Co-V decreases from the value of FeCo bulk to a value less than the lattice constant of Rh (*a*_Rh_/√2). In the out-of-plane XRD patterns, as *y* increases, the Fe-Co-V peak shifts from the bcc-FeCo(002) peak position at approximately 65° to the vicinity of the fcc-FeCo(002) peak position at approximately 50°. This peak shift indicates that the lattice constant *c* of the Fe-Co-V increases from the value of bulk bcc-FeCo to a value close to the lattice constant of fcc-FeCo.

Figure 1b shows the *y* dependence of the axial ratio *c*/*a* of the Rh/(Fe_0.5_Co_0.5_)_100−*y*_V_*y*_ (*t* = 20 nm) films. The lattice constants *a* and *c* were calculated from the in-plane and out-of-plane XRD patterns, respectively. The error bars were calculated from the full widths at half maximum of the XRD peaks. In the range of 0 ≤ *y* ≤ 15 at%, a bcc structure with *c*/*a* ≈ 1.00 is formed, and in the range of 18 ≤ *y* ≤ 50 at%, a bct structure with the ideal value of *c*/*a* ≈ 1.25 is formed. The bcc‒bct transformation occurred discontinuously with respect to the V composition. For the sample with *y* = 50 at% in Fig. 1a, the peaks derived from Fe-Co-V are weaker, which suggests that amorphisation may have progressed. This is currently under investigation.

**Fig. 2:**
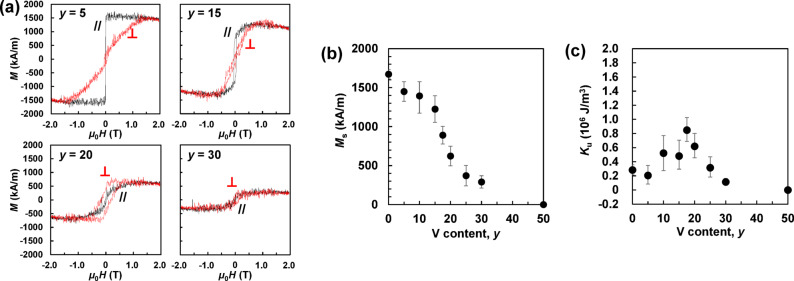
(**a**) Magnetisation curves, *y* dependence of (**d**) *M*_s_ and (**e**) *K*_u_ of the MgO sub./Rh (20 nm)/(Fe_0.5_Co_0.5_)_100−*y*_V_*y*_ (20 nm)/SiO_2_ (5 nm) films.

Figure 2a shows the magnetisation curves of the Rh/(Fe_0.5_Co_0.5_)_100−*y*_V_*y*_ (*t* = 20 nm) films. In the range of 0 ≤ *y* ≤ 15 at%, where the bcc structure is formed in Fig. 1, the magnetic easy axis is along the in-plane direction. In the range of 18 ≤ *y* ≤ 30 at%, where the bct structure of *c*/*a* ≈ 1.25 is formed, because the perpendicular magnetisation curve (⟂) saturates at a lower magnetic field than the parallel magnetisation curve (//), it is determined that the magnetic easy axis is in the perpendicular direction, and a relatively high coercivity of approximately 0.2 T is obtained. An exchange bias was confirmed at 30 K in the film with *y* = 25 (data not shown here); therefore, it is considered that an antiferromagnetic phase formed by V distribution in part of the film can become a pinning site for domain-wall motion, resulting in a relatively high coercivity.

Figures 2b,c show the *y* dependence of the saturation magnetisation *M*_s_ and the uniaxial magnetic anisotropy constant *K*_u_ of the Rh/(Fe_0.5_Co_0.5_)_100−*y*_V_*y*_ (*t* = 20 nm) films, respectively. The error bars were calculated from the noise widths of the magnetisation curves recorded by a vibrating-sample magnetometer (VSM). *K*_u_ was calculated from the difference in area between the perpendicular and in-plane magnetisation curves. In the case of an in-plane magnetisation film, the component of the shape magnetic anisotropy is subtracted from the difference in area, and in the case of a perpendicular magnetisation film, the component of the shape magnetic anisotropy is added to the difference in area. Following convention, perpendicular magnetic anisotropy was defined as positive. As *y* increased, *M*_s_ decreased faster than monotonically, and the *M*_s_-value reached to ~ 0 kA/m for the film with *y* = 50. This could be attributed to the antiferromagnetism, as mentioned above. On the other hand, *K*_u_ reaches a maximum value of ~ 10^6^ J/m^3^ at *y* ≈ 18‒20, where the bct structure with *c*/*a* ≈ 1.25 is formed as a main or sub-phase, and the perpendicular magnetisation film, whose perpendicular magnetisation curve saturates at a magnetic field lower than the in-plane magnetisation curve, is obtained.

From these results, it can be concluded that the optimal V content *y* for bct formation and high *K*_u_ is approximately 20 at%.

### Crystal structure of Rh/(Fe_0.5_Co_0.5_)_80_V_20_ (*t* nm)

**Fig. 3:**
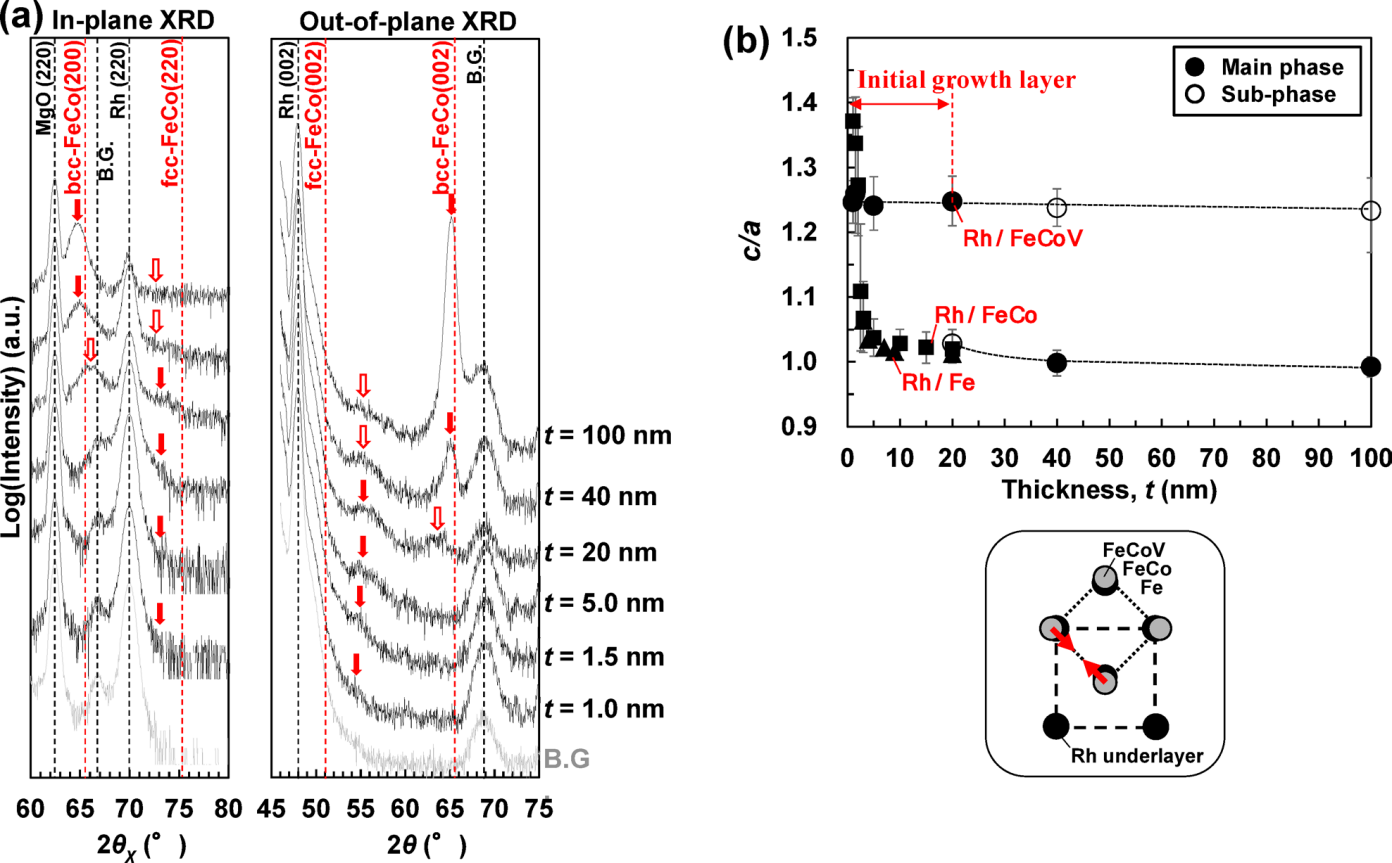
In-plane and out-of-plane XRD patterns of (**a**) MgO sub./Rh (20 nm)/(Fe_0.5_Co_0.5_)_80_V_20_ (*t* nm)/SiO_2_ (5 nm). (**b**) *t* dependence of *c*/*a* of MgO sub./Rh (20 nm)/Fe (*t* nm)/SiO_2_ (5 nm), MgO sub./Rh (20 nm)/Fe_50_Co_50_ (*t* nm)/SiO_2_ (5 nm), and MgO sub./Rh (20 nm)/(Fe_0.5_Co_0.5_)_80_V_20_ (*t* nm)/SiO_2_ (5 nm). The inset shows a top view of the crystal orientation relationship between the Rh underlayer and the Fe, FeCo, and FeCoV films.

The optimal range of film thickness *t* for bct formation was investigated by fixing the V content to *y* = 20 at%, which formed bct with *c*/*a* ≈ 1.25 in Fig. 1. In the in-plane and out-of-plane XRD patterns of the Rh/(Fe_0.5_Co_0.5_)_80_V_20_ (*t* = 20 nm) films in Fig. 3a, only the main-phase peak (red solid arrow) is observed in the thin film region (*t* ≤ 5.0 nm), but at *t* = 20 nm and above, two types of peaks, the main-phase peak and the sub-phase peak (red hollow arrow), are observed. In the out-of-plane XRD patterns, these two peaks are observed at approximately 55° and 65°, and no peaks are observed at other angles. In the thin region of *t* ≤ 5.0 nm, only the peak at around 55°, which indicates the formation of bct phase, is observed. This peak corresponds to the initial growth layer, as described below. In the medium-thickness region of *t* = 20 nm, the bct phase (approximately 55°) is formed as the main phase, and the bcc phase, which is in thermal equilibrium, begins to form a sub-phase (around 65°). In the thick region of *t* ≥ 40 nm, the bcc phase becomes the main phase (around 65°), and the sub-phase peak (initial growth layer) is also observed at around 55°.

Here, we focus on the peaks at approximately 55°, which indicate the formation of the bct phase in the out-of-plane XRD pattern. The intensity of these peaks increases and saturates with increasing *t*, and it becomes constant in the thick region of *t* ≥ 20 nm. The bct phase originating from these peaks is considered to be the initial growth layer (i.e. the phase formed at the interface with the Rh underlayer in the early stages of film formation). The maximum thickness of the initial growth layer was estimated to be approximately 20 nm.

Figure 3b shows the *t* dependence of the *c*/*a* ratios of the Fe, Fe-Co, and Fe-Co-V films deposited on the Rh underlayer. The inset shows a top view of the crystal orientation relationship between the Rh underlayer and the Fe-based alloy films. For example, the lattice mismatch between the Rh and the Fe_50_Co_50_ is (*a*_FeCo_ − *a*_Rh_/√2)/*a*_FeCo_ ≈ 0.05. In the ultrathin region (*t* ≤ 2 nm), a bct structure with 1.00 < *c*/*a* < 1.41 was formed in all the Fe, Fe-Co, and Fe-Co-V films. However, in the thicker region (*t* > 2 nm), the bct structure rapidly transformed back into a bcc structure (in the thermal equilibrium state) in the Fe and Fe-Co films. In contrast, for the Fe-Co-V films, the bct structure was formed as the main phase up to the medium-thickness region of *t* ≤ 20 nm. This result indicates that FeCo, which forms the bcc phase in the thermal equilibrium state, easily transforms into bct when V is added under compressive stress owing to the lattice mismatch with the Rh underlayer. In other words, the added V lowers the energy needed to change the lattice constant of FeCo.

### Binding energy, crystal structure, and magnetic properties of Rh/((Fe_0.5_Co_0.5_)_0.8_V_0.2_)_100−*x*_N_*x*_

**Fig. 4:**
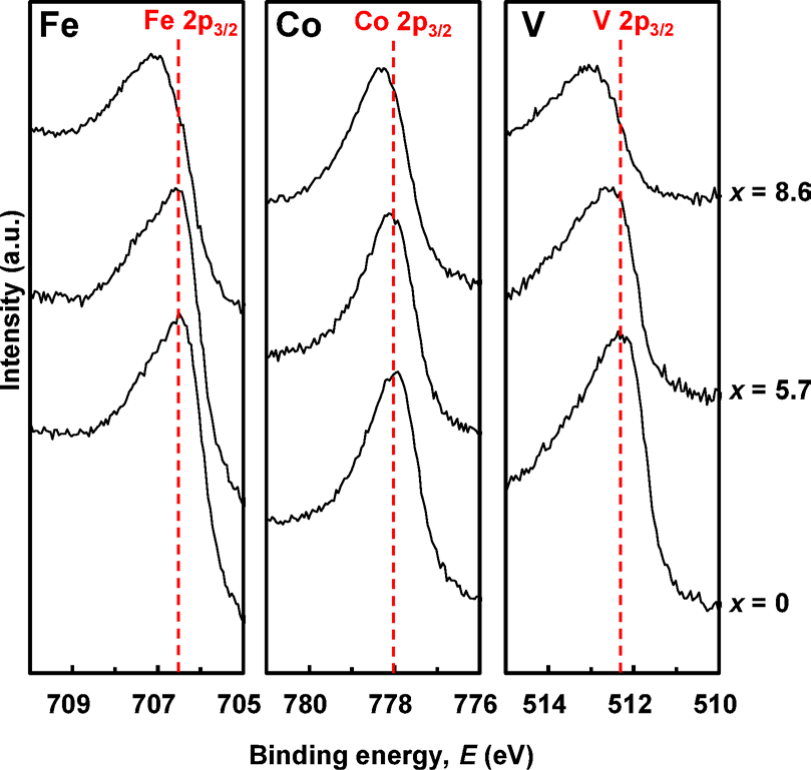
XPS spectra of Fe, Co, and V atoms for MgO sub./Rh (20 nm)/((Fe_0.5_Co_0.5_)_0.8_V_0.2_)_100−*x*_N_*x*_ (20 nm)/SiO_2_ (5 nm).

Next, we investigated the effect of nitridation on bct formation in Fe-Co-V. The amount of V was fixed at *y* = 20 at%, which formed the bct structure with *c*/*a* ≈ 1.25 in Fig. 1. Figure 4 shows the X-ray photoelectron spectroscopy (XPS) spectra of the Rh/((Fe_0.5_Co_0.5_)_0.8_V_0.2_)_100−*x*_N_*x*_ (*t* = 20 nm) films. When the amount of N (*x*) was increased from 0 to 5.7, the peak positions of the Fe and Co atoms hardly changed, but that of the V atom shifted to the left approximately 0.36 eV. It is interpreted that in the Fe-Co-V-N film, the V atoms are mainly bonded to the N atoms, whereas the Fe and Co atoms are hardly bonded to the N atoms. For the sample with *x* = 8.6, the peak positions of the Fe, Co, and V atoms all shifted to the left approximately 0.54, 0.26, and 0.64 eV, respectively; in particular, those of the V and Fe atoms shifted significantly. It is inferred that the Fe, Co, and V atoms were all bonded to N atoms, but V and Fe atoms were preferentially bonded to N atoms.

It has been reported that for Fe-Co-N films (*t* = 20 nm) without V addition, a discontinuous transformation from bcc to fcc occurs with increasing N addition, and the bct phase is not formed^15^. In contrast, for Fe-Co-V-N films, the bct phase is formed not only on the Rh underlayer but also on the amorphous SiO_2_ substrate^13–15,17,18^. These reports do not contradict the consideration that V lowers the energy required for the lattice constant of FeCo to change, as mentioned in Fig. 3. From these results, it is considered that when Fe-Co without V is nitrided, N atoms mainly bond to Fe atoms; however, because there are no V atoms, lattice deformation is difficult, and therefore, a discontinuous bcc‒fcc transformation occurs. In contrast, when the Fe-Co-V is nitrided, the V atoms are first nitrided, and the lattice constant easily changes to that of a bct structure, owing to the N atoms. Subsequently, when excess N was supplied, both the V and Fe atoms were nitrided and eventually transformed into the fcc structure.

**Fig. 5:**
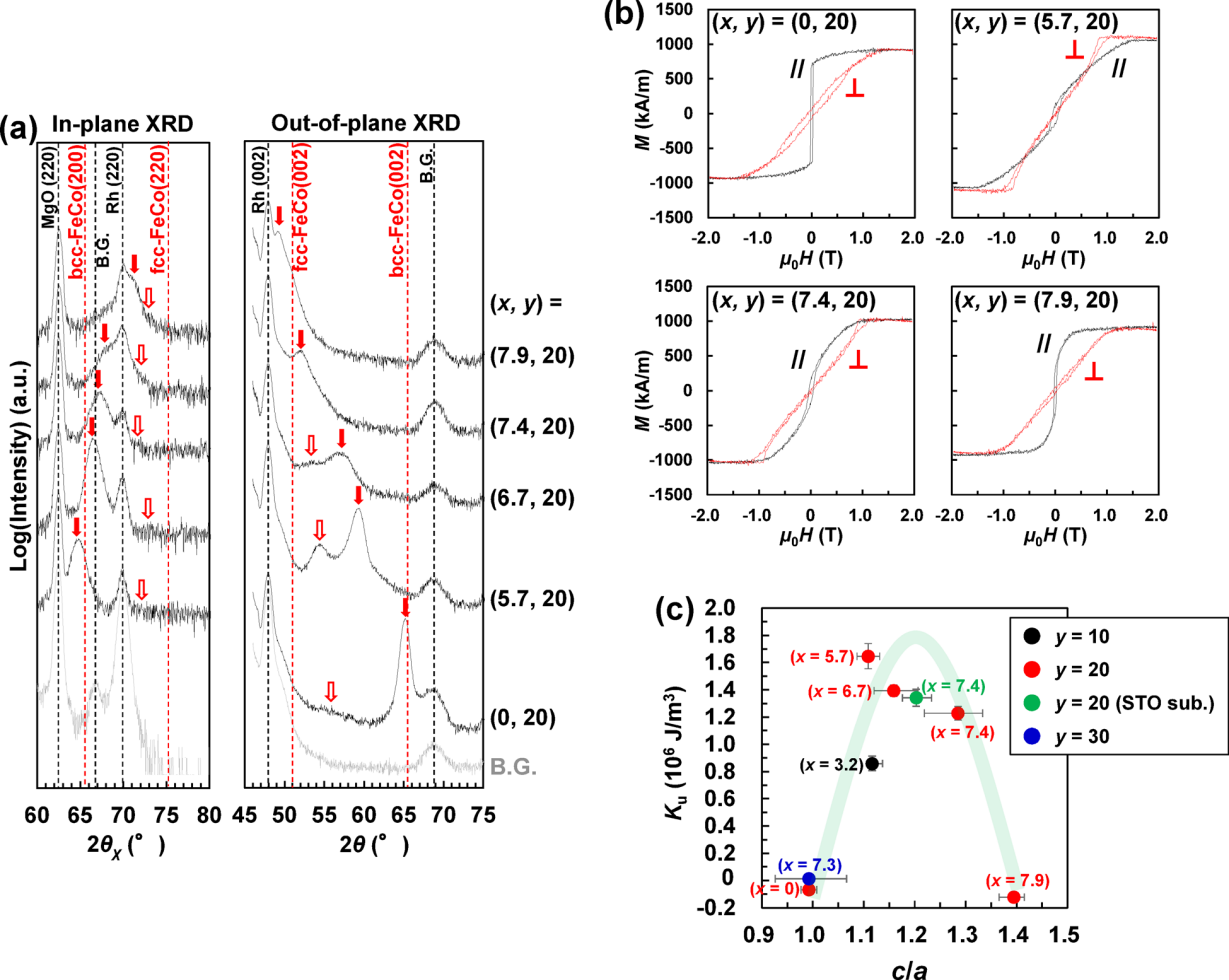
(**a**) In-plane and out-of-plane XRD patterns, (**b**) magnetisation curves, (**c**) *K*_u_ as a function of *c*/*a* for MgO sub./Rh (20 nm)/((Fe_0.5_Co_0.5_)_100−y_V_y_)_(100−x)/100_N_x_ (100 nm)/SiO_2_ (5 nm).

Figure 5a shows the in-plane and out-of-plane XRD patterns of Rh/((Fe_0.5_Co_0.5_)_0.8_V_0.2_)_100−*x*_N_*x*_ (*t* = 100 nm) thick films. In the out-of-plane XRD pattern of the (*x*, *y*) = (0, 20) film without N addition, a bcc peak was observed at approximately 65° as the main-phase peak, and a bct peak was observed at approximately 55° as the sub-phase peak. This bct sub-phase was the initial growth layer, as shown in Fig. 3b, and its thickness was estimated to be ~ 20 nm. With an increase in *x*, the main phase changed from bcc to bct and then to fcc. For the (*x*, *y*) = (5.7, 20) film, a large bct peak of the main phase was observed at approximately 60° in the out-of-plane XRD pattern. For the (*x*, *y*) = (7.9, 20) film, the fcc peak of the main phase was observed at approximately 50°.

Figure 5b shows the magnetisation curves of the Rh/((Fe_0.5_Co_0.5_)_0.8_V_0.2_)_100−*x*_N_*x*_ (*t* = 100 nm) thick films. Atomic force microscopy confirmed that the film surfaces of all the samples were continuous (i.e. the crystal grains were not spherical) (data not shown here). Therefore, it is appropriate to use the demagnetisation factor (~ 1.0) of a plate-shaped sample to evaluate their magnetic properties^1^. In the films with (*x*, *y*) = (0, 20) and (7.9, 20), whose main phase is bcc and the magnetic easy axis is in-plane, the saturation field (i.e. inflection point of magnetisation curve) of the perpendicular magnetisation curve is almost the same as the demagnetisation field (*µ*_0_*M*_s_ ≈ 1.1 T). Therefore, the *K*_u_ values of these samples were estimated to be approximately zero. In contrast, in films with (*x*, *y*) = (5.7, 20) and (7.4, 20), in which the main phase is the bct phase, the perpendicular magnetisation curve is saturated at a magnetic field lower than the demagnetisation field; therefore, it is estimated that perpendicular magnetic anisotropy is induced. In particular, the sample with (*x*, *y*) = (5.7, 20), for which the largest bct peak is observed in Fig. 5a, is a perpendicular magnetisation film.

Figure 5c shows the correlation between *c*/*a* and *K*_u_ estimated from the magnetisation curves in Fig. 5b. The *K*_u_ was calculated from the difference in area between the perpendicular and in-plane magnetisation curves. The data for a film with *y* = 10 (black circle) and *y* = 30 (blue circle) are also plotted. A mountain-like trend (green line in the figure) was observed, with a maximum peak at approximately *c*/*a* = 1.2. A high *K*_u_ of ~ 10^6^ J/m^3^ was obtained for films with (*x*, *y*) = (5.7, 20), (6.7, 20), and (7.4, 20), whose main phase was bct. Therefore, the optimal *x* and *y* are approximately 5.7 and 20, respectively, and the optimal *x* predicted from the vertex of the parabola is approximately 7.0.

Data (green circle) for SrTiO (STO) sub./((Fe_0.5_Co_0.5_)_0.8_V_0.2_)_100−*x*_N_*x*_ (*x* = 7.4 at%, *t* = 100 nm) deposited on an STO substrate (whose lattice constant is close to that of FeCo) is also plotted in the figure. Crystal structure analysis and magnetic measurements for this sample revealed that a bct single phase with *c*/*a* ≈ 1.25 was formed, and a high *K*_u_ of 1.3 × 10^6^ J/m^3^ was obtained. Thus, the formed bct phase appeared to be stable, regardless of the lattice constant of the underlayer.

These findings suggest that the combined addition of V and N based on method (ii) is useful for obtaining a bct phase in samples deposited on amorphous substrates, thick films, bulk samples, etc., in which it is difficult to utilise the compressive stress derived from the lattice mismatch using method (i).

## Discussion

**Fig. 6:**
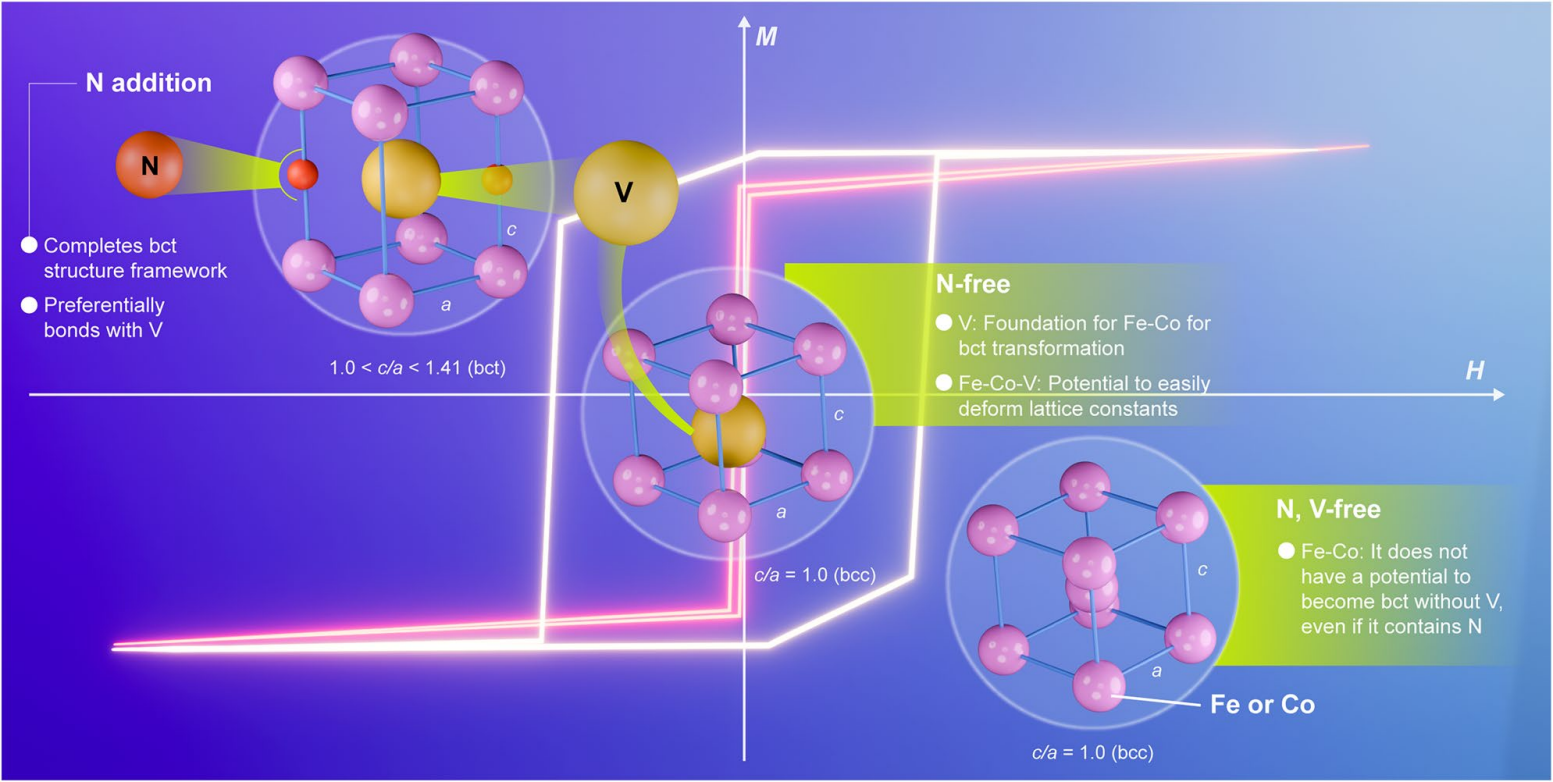
Schematic image of the roles of V and N in the bct formation in FeCo.

First, we considered the role of V in the bct formation of FeCo. As shown in Figs. 1, 2 and 3 and N-free (Fe_0.5_Co_0.5_)_100−*y*_V_*y*_ (*t* nm) was epitaxially grown on the (001)-oriented Rh underlayer. The lattice constant of the Rh (*a*_Rh_/√2) was smaller than that of the Fe_50_Co_50_ (*a*_FeCo_); thus, the FeCo lattice was always under compressive stress (see Fig. 3(b)). In the Fe_50_Co_50_ (*y* = 0) film, a bcc single phase in a thermal equilibrium state was formed. By increasing *y*, a bct structure with *c*/*a* ≈ 1.25 was formed at *y* ≥ 18, and the bct phase formed in the medium-thickness region (*t* ≤ 20 nm) was the initial growth layer due to the lattice mismatch between the Rh and the Fe-Co-V. The lattice volume of the Fe-Co-V was almost constant, exhibiting no correlation with *y* (data not shown). In addition, for (Fe_0.5_Co_0.5_)_100−*y*_V_*y*_ deposited on an STO substrate, whose lattice constant was close to that of the Fe-Co-V, only a bcc structure was formed regardless of the amount of *y*. From the above results, it is considered that V facilitates the change in the lattice constant, and when compressive stress is added, the structure easily changes from bcc to bct. In other words, adding only V does not affect the crystal structure as shown in Fig. 6, but some type of stress must act on the lattice changes. Therefore, we conclude that V lowers the energy needed to change the lattice constant of FeCo.

Next, we examined the role of N in bct formation. In the cases of Figs. 4 and 5, the Rh/((Fe_0.5_Co_0.5_)_0.8_V_0.2_)_100−*x*_N_*x*_ (*t* nm) was prepared up to the thick region of *t* ≤ 100 nm. The bcc phase was the main phase when no N was added (*x* = 0). With an increase in *x*, the lattice constant *c* increased, and the main phase changed from bcc to bct and then to fcc. The lattice volume of Fe-Co-V-N increased with *x*. This tendency differed significantly from that of N-free Fe-Co-V. In the Fe-Co-V-N, the N atoms are considered to exist along the *c*-axis as shown in Fig. 6 and apply stress in a direction that elongates the *c*-axis with little change in the *a*-axis. Because the N atoms preferentially bond to the V atoms, it is considered that the V and N atoms are in a cluster-like state at the atomic scale (not indicating macroscale segregation of the V-N phase) in the Fe-Co-V-N lattice.

### Summary

We investigated the optimal V and N contents for the bct transformation of FeCo and found that they were approximately 20 at% V and 5.7 at% N. We also found that these optimal compositions were effective even in the thick-film region of ~ 100 nm.

The role of V in the bct transformation is to lower the energy needed to change the lattice constant of FeCo and attract N atoms. The role of N is to apply stress in a direction that elongates the *c*-axis by existing in the Fe-Co-V lattice, changing the structure to bct.

From the above, we consider that the combined addition of V and N to FeCo has the effect of stabilising or metastabilising the bct phase and is useful for producing FeCo-based permanent magnets.

## Methods

Using an ultrahigh-vacuum (~ 10^−7^ Pa) magnetron co-sputtering system, a Rh underlayer (*t* = 20 nm) was deposited on an MgO (100) single-crystal substrate at a substrate heating temperature of 573 K, followed by the deposition of Fe-Co-V or Fe-Co-V-N (*t* nm) at 473 K. Then, an SiO_2_ (*t* = 5 nm) cap layer was deposited on top at 293 K. The film compositions were as follows: MgO sub./Rh (20 nm)/(Fe_0.5_Co_0.5_)_100−*y*_V_*y*_ (*t* nm)/SiO_2_ (5 nm) and MgO sub./Rh (20 nm)/((Fe_0.5_Co_0.5_)_100−*y*_V_*y*_)_100−*x*_N_*x*_ (*t* nm)/SiO_2_ (5 nm).

The composition ratio of Fe-Co-V was determined by controlling the input power of each Fe, Co, and V single-substance target and analysing the composition using an electron probe microanalyser and XPS. The amount of N atoms was determined by controlling the mixing ratio of the Ar and N_2_ gases (N_2_/(Ar + N_2_)) during sputtering and analysing the composition using XPS. XRD analysis (using Cu*K*_α_ radiation) in the in-plane and out-of-plane modes was used for crystal structure analysis. A VSM (with a maximum field of 2.0 T) was used to evaluate the magnetic properties.

## Data Availability

The datasets used and/or analysed during the current study available from the corresponding author on reasonable request.
